# Lumbar puncture resulting in lumbar artery perforation and hypotension: A case report

**DOI:** 10.1016/j.radcr.2025.04.016

**Published:** 2025-05-16

**Authors:** Anthony D’Angelo, Mbinui Ghogomu, Zahra Akhtar, Hasan Khan

**Affiliations:** a John Sealy School of Medicine, The University of Texas Medical Branch, Galveston, TX, USA; b Department of Radiology, The University of Texas Medical Branch, Galveston, TX, USA

**Keywords:** Lumbar puncture, Case report, Embolization, Retroperitoneal hemorrhage, Hypotension

## Abstract

Lumbar puncture (LP) is a very common procedure that can rarely lead to complications such as headache or spinal hematoma. This report presents the case of a 59-year-old female who underwent LP and subsequently developed a retroperitoneal hematoma leading to hemorrhagic shock, requiring emergent embolization by interventional radiology. Life-threatening bleeding is a very rare complication of LP but should be kept in mind especially for patients with large body habitus who undergo multiple unsuccessful bedside attempts. In this case, the LP needle missed the spinal canal and pierced the L4 lumbar artery. We present this case to uncover potential factors that may have contributed to the development of such hemorrhage and to elaborate the steps of care to control such devastating hemorrhage.

## Introduction

A lumbar puncture (LP) is a common low risk procedure that is used to obtain cerebrospinal fluid (CSF) for diagnostic purposes as well as for therapeutic purposes such as intracranial pressure relief, anesthesia, and medication administration [1,2]. While usually a safe procedure, LP can result in various complications such as a postlumbar puncture headache, back pain, discomfort at the injection site, or transient paresthesia. Most complications are generally self-limiting, and severe or fatal side effects such as infection, spinal hematoma, or brain herniation are uncommon [1,3]. A rare complication of LP is bleeding outside of the spinal column with only a few instances cited in the literature [1]. This case report discusses a 59-year-old female who underwent a bedside lumbar puncture and developed a retroperitoneal hematoma along with hemorrhagic shock, requiring emergency intravascular embolization.

## Case presentation

**Fig. 1:**
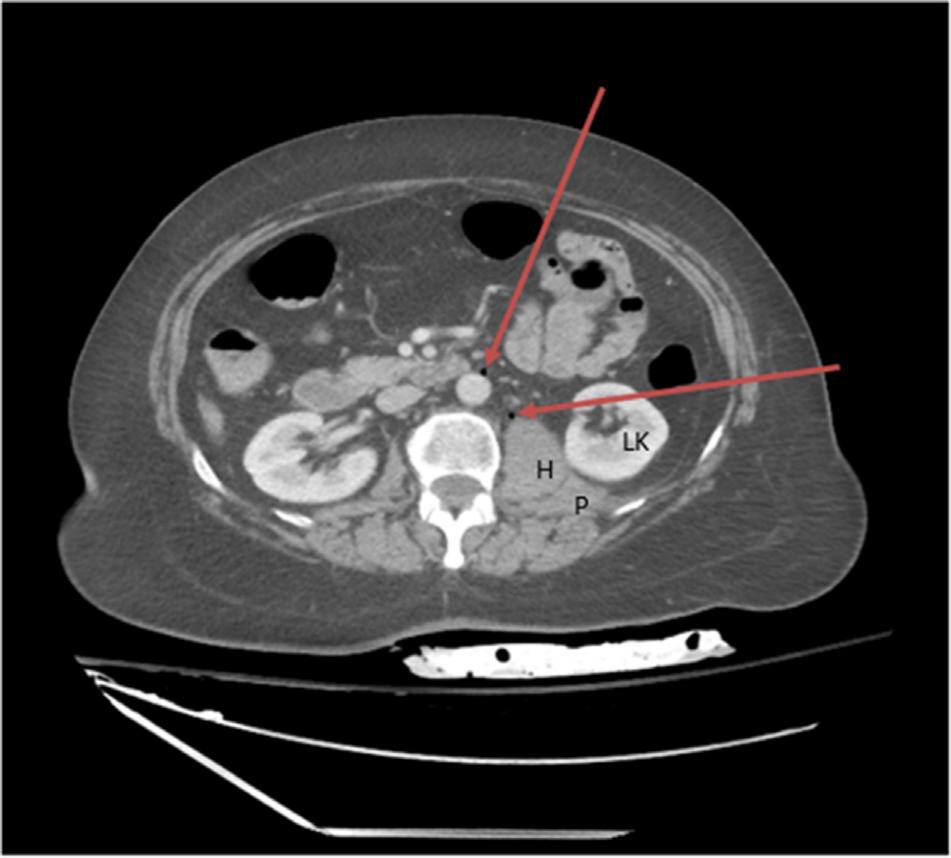
Left psoas muscle (P) with a small left retroperitoneal hematoma (H). Foci of air (arrows) were noted along the anterior aspect of the left psoas muscle as well as the infrarenal abdominal aorta.

**Fig. 2:**
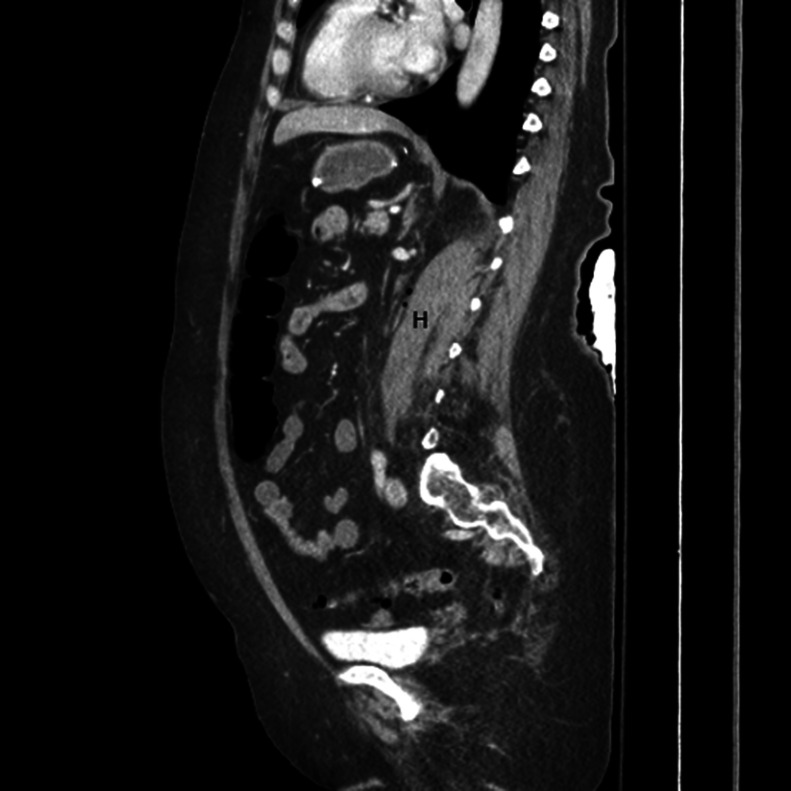
Sagittal view of left psoas hematoma (H).

A 59-year-old woman with a past medical history significant for hypertension and obesity was hospitalized due to 3 weeks of bilateral ascending lower extremity numbness and weakness. Due to concerns about possible Guillain-Barre Syndrome (GBS) or Acute Inflammatory Demyelinating Neuropathy (AIDN), a bedside LP was performed to collect CSF samples and the patient was started on intravenous immunoglobulin (IVIG). The initial LP was unsuccessful in collecting samples, and the patient was scheduled to have interventional radiology (IR) attempt LP again the next day. During the night, the patient’s blood pressure (BP) dropped to 77/51 with tachycardia and hemoglobin dropped from 15.0 to 13.0. CT abdomen and pelvis revealed a retroperitoneal bleed and hematoma on the left psoas muscle (Figs. 1, 2), resulting in the patient being transferred to the neuroscience critical care unit (NCCU).

**Fig. 3:**
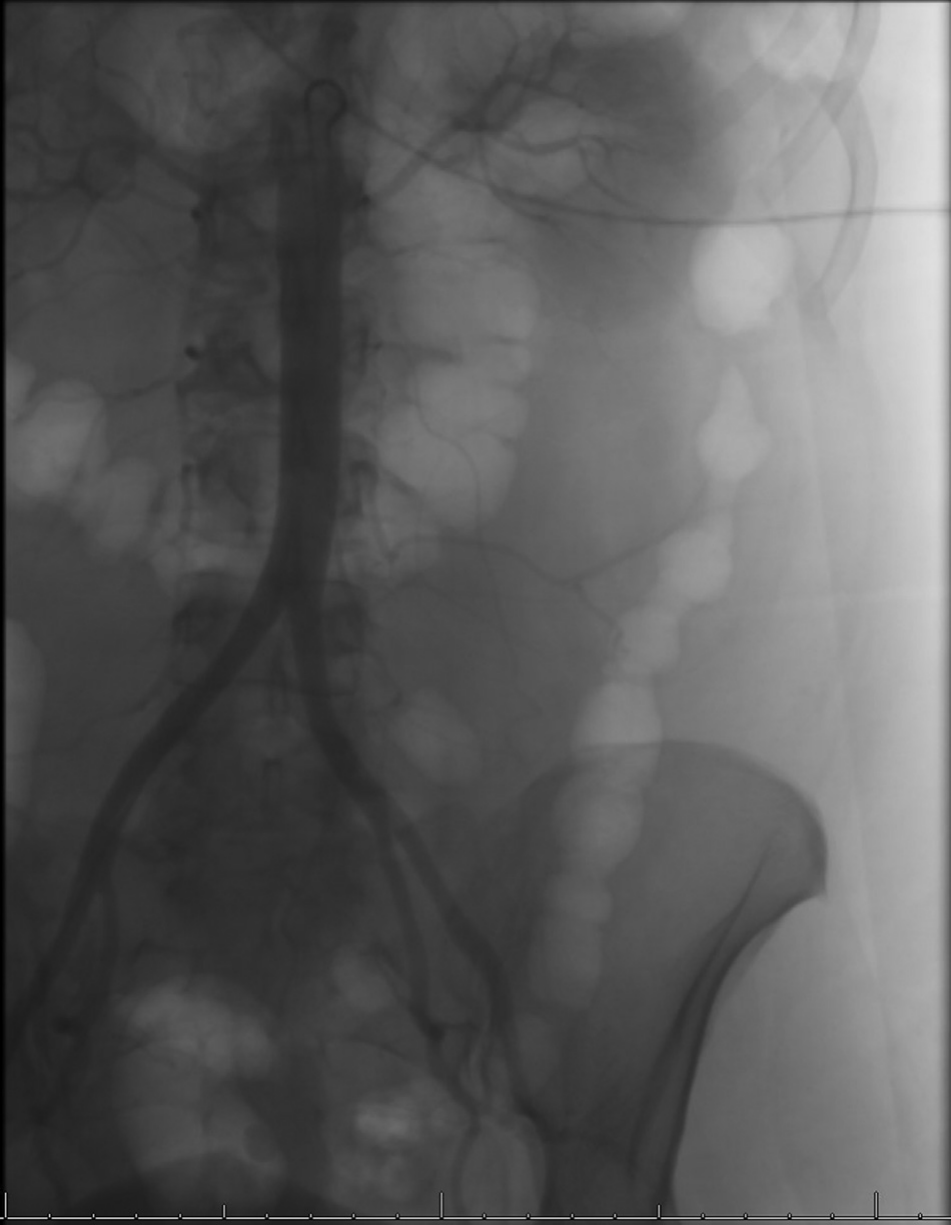
Initial abdominal aortogram showing renal arteries, iliac arteries, and lumbar arteries.

**Fig. 4:**
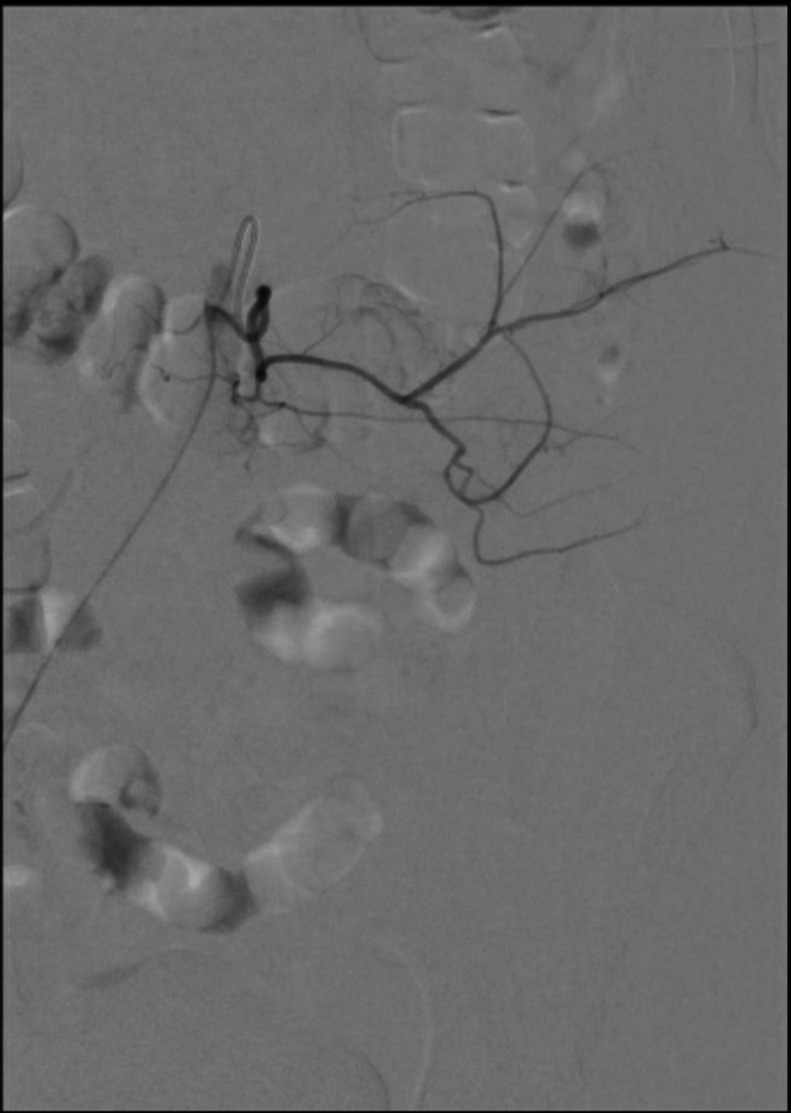
Left L2 arteriogram, which showed no signs of vasospasm or extravasation.

**Fig. 5:**
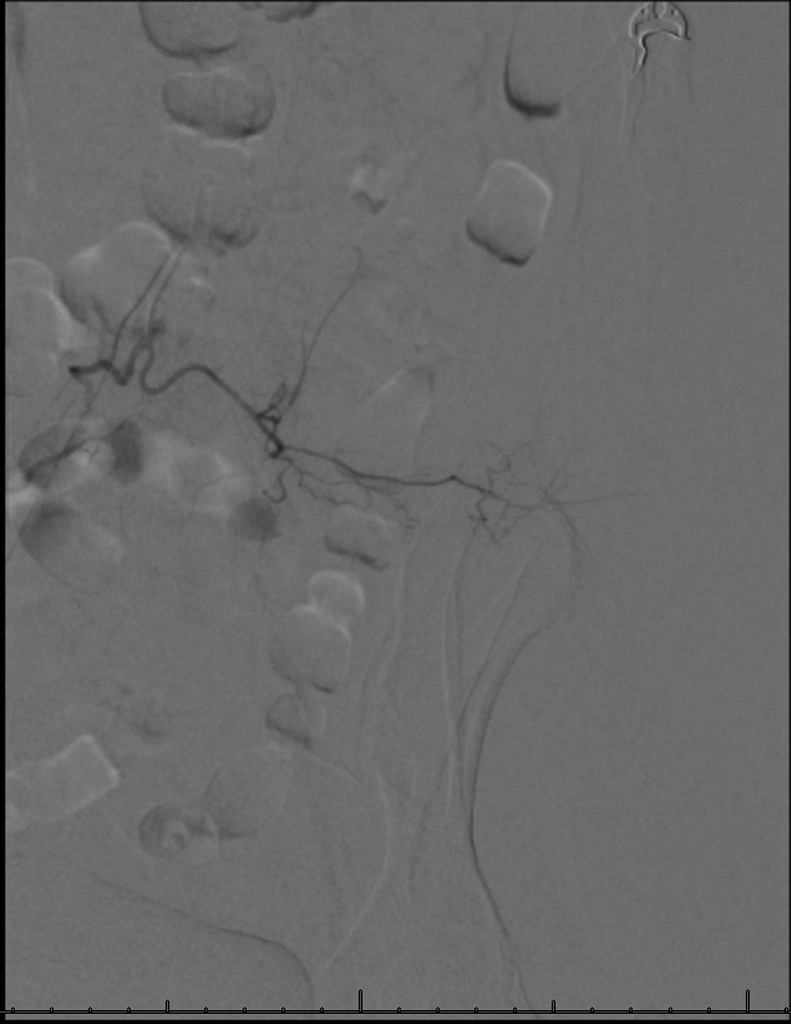
Left L4 arteriogram with signs of reduced blood flow, constantly narrowed arteries, and gradual tapering at more distal vessels, consistent with vasospasm.

**Fig. 6:**
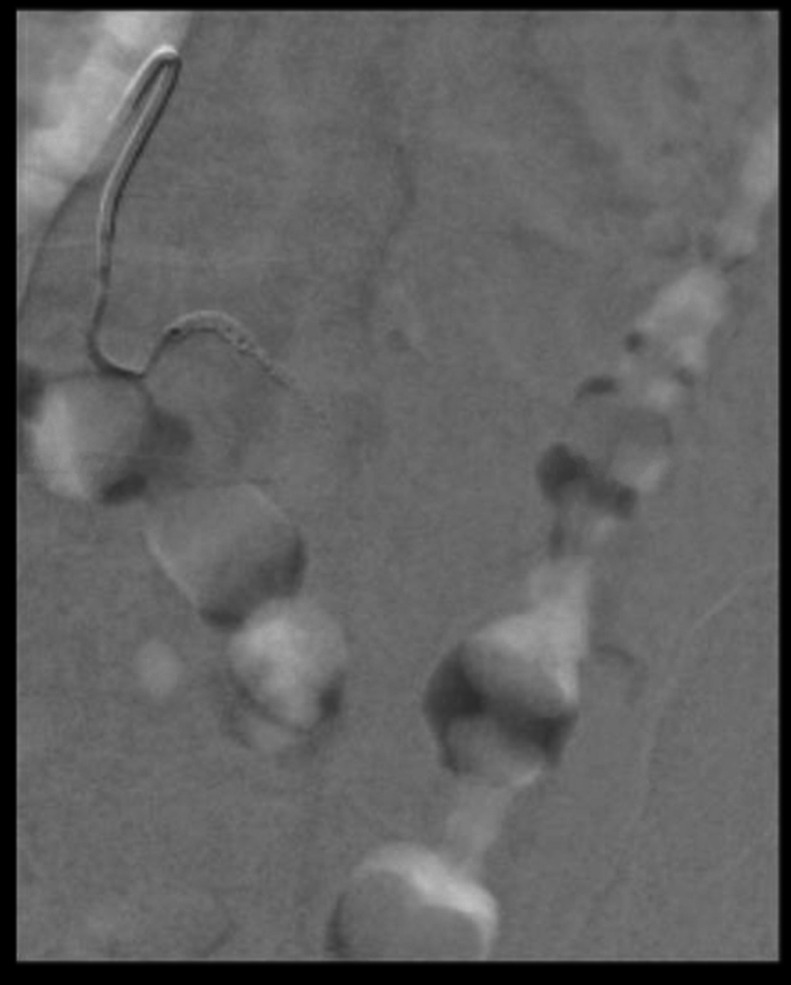
Postcoil left L4 arteriogram showing successful embolization.

At this point, IR was consulted to find and embolize the source of the bleeding. The patient was brought to the IR suite and the right common femoral artery was accessed with a 21 Gauge micropuncture kit using ultrasound guidance. The access was upsized to a 5 Fr, 10 cm sheath using Seldinger’s technique. A 5 Fr reverse curve catheter was advanced through the right common femoral artery to the abdominal aorta (Fig. 3), and the left lumbar arteries L1-L4 were individually selected and digital subtraction angiogram (DSA) performed to look for signs of hemorrhage (Fig. 4). While no contrast active extravasation was detected, the left L4 lumbar vessel showed vasospasm, a hemodynamic vessel response to recent bleeding (Fig. 5). After selecting the left L4 lumbar artery with a 2.4 Fr microcatheter, embolization was performed with 3 microcoils followed by Gelfoam slurry. Postembolization DSA showed successful hemostasis (Fig. 6). No significant hemodynamic events occurred for the remainder of the patient’s hospitalization. The patient completed IVIG therapy and was discharged after 5 additional days of hospitalization with significantly improved lower extremity neurological function and planned outpatient follow-up.

## Discussion

Lumbar puncture involves accessing the subarachnoid space in the spinal cord, usually below the L2 vertebra, in order to get a sample of cerebrospinal fluid (CSF) or to relieve intracranial pressure [4]. Indications include central nervous system (CNS) infections, presence of stroke or neoplasm, and relieving ICP [4]. In adults, access is done below the L2 vertebra to reduce the risk of causing damage to the spinal cord, which terminates above L2 at the conus medullaris and becomes multiple smaller nerves in the cauda equina [4]. To perform a lumbar puncture, the patient is placed in the recumbent position with knees and neck flexed. The insertion point is sterilely prepped, and a needle is put through between L3 and L4, passing through the skin, subcutaneous tissue, supraspinous ligament, interspinous ligament, ligamentum flavum, epidural space, dura mater, and arachnoid mater until reaching the subarachnoid space [4]. Upon entering this space, a “pop” will usually be heard or felt, and passive drainage of CSF can be done. The most common postprocedural complication for lumbar puncture is headache, believed to be due to CSF leaking through the Dural puncture. Headaches usually are self-limited to 2 weeks, are positional in occurrence, and usually result in neck stiffness. A less common complication of lumbar puncture is a spinal hematoma, which results in symptoms of radiating back pain, anal sphincter dysfunction, and new-onset sensory disturbances [4]. Among patients receiving lumbar puncture, about 2% of patients on anticoagulants may have a spinal hematoma, with substantially fewer seen in those not on anticoagulants [2].

Retroperitoneal hematomas can be life-threatening due to lack of symptoms until significant blood loss has already occurred. They can be classified as traumatic or nontraumatic depending on etiology, with nontraumatic being further classified as spontaneous or nonspontaneous/iatrogenic [5]. While the incidence of iatrogenic retroperitoneal hematoma is low at 0.06%, there is 3.5 times increase in 30 day mortality. Risk factors include arterial punctures above the inguinal ligament, female sex, and being treated with antiplatelet agents or warfarin. Management includes management of blood loss, CT scan to determine the source of the bleed, and either surgery or IR embolization to stop the bleed. Anticoagulation should also be stopped [5].

**Fig. 7:**
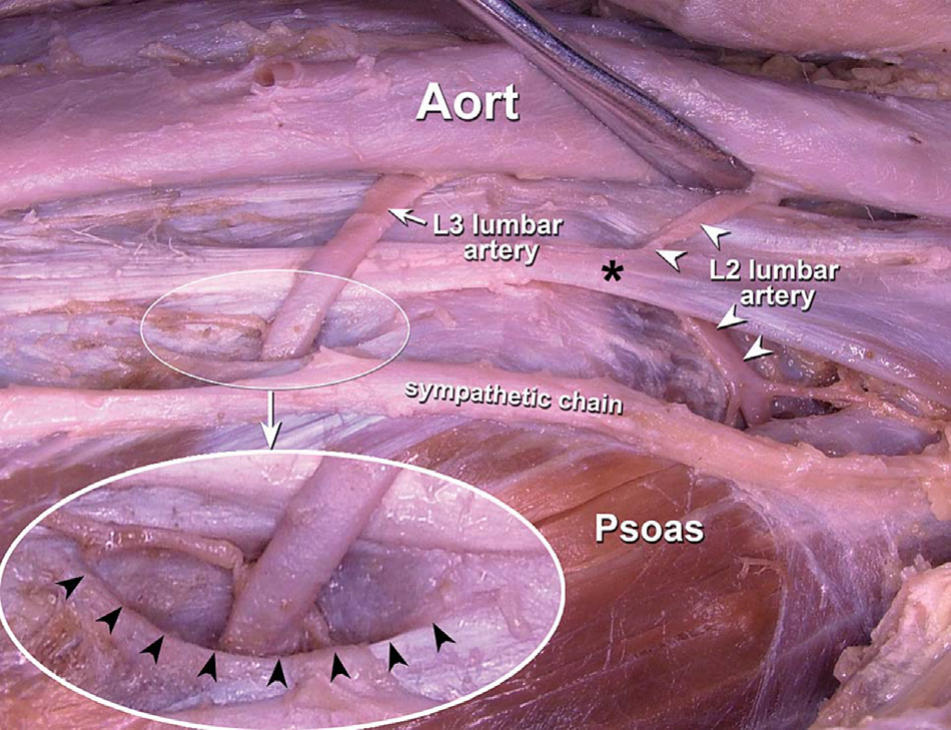
Anatomical cadaver dissection showing relative locations of lumbar arteries in the retroperitoneal space. In this case, the L2 and L3 arteries can be seen branching off the aorta, crossing superficial to the diaphragmatic crus (*), and then running deep to the sympathetic chain and psoas muscle [7].

**Fig. 8:**
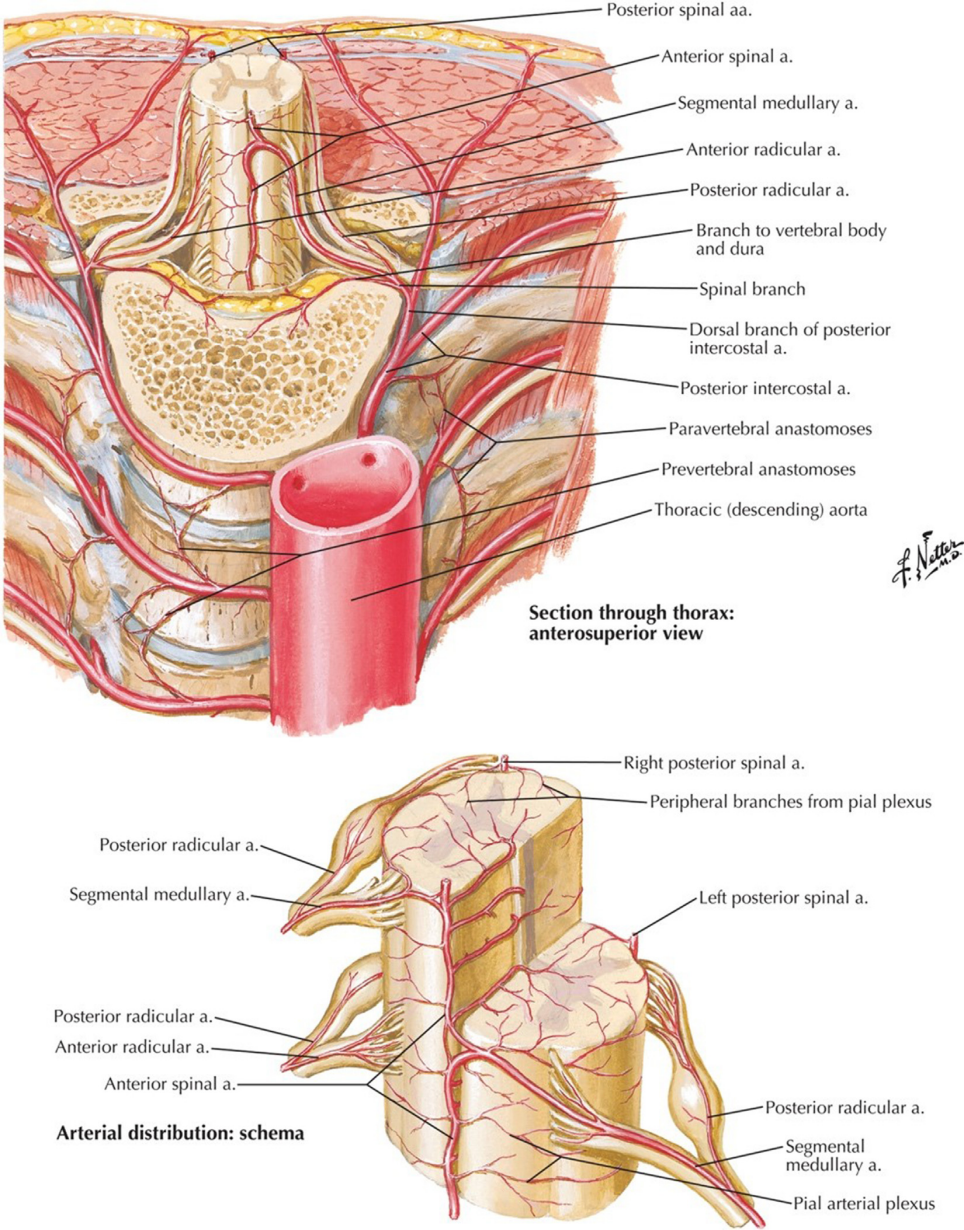
Anatomical images showing the general layout of spinal arteries. Here, a posterior intercostal artery comes off the aorta, branching to become a segmental spinal artery. The other portion continues around inferior to the rib. Lumbar arteries follow a similar path [8].

Retroperitoneal hematomas due to lumbar puncture are rare, but cases have been reported. Horani et al describe a case of a 75-year-old woman who, after several unsuccessful lumbar punctures, had a left psoas hematoma on imaging, with IR embolization at the L3-L4 left lumbar artery successfully controlling the bleeding [1]. Similarly, Oladeji et al discusses IR embolization of the right L2 lumbar artery in a patient who presented a week after LP with right sided abdominal back pain radiating down the right leg. In this case, the patient required 3 attempts at LP, with the presence of blood suggesting a traumatic tap and abdominal CT before IR embolization showing a right psoas hematoma [2]. Looking at the anatomy around the lumbar arteries, they can be seen to run retroperitoneal and close to both the psoas muscle before going deep to reach the spinal cord and other dorsal structures (Figs. 7, 8). In these cases, accidental artery puncture and retroperitoneal hemorrhage can occur, albeit rarely.

Various IR embolization techniques are available. Particles such as resorbable Gelfoam or nonresorbable microbeads are popular for occluding smaller vessels. Liquid embolics such as Onyx are highly potent in causing occlusion to the capillary level, but require care due the risk of nontarget embolization. Coils, which cause thrombosis within the target vessel, are very popular especially for acute cases in which embolization must be done rapidly. Lastly, vascular plugs can be used for larger blood vessels that would normally require many coils to fully embolize [6].

This patient who received LP at our institution had a complication of retroperitoneal hematoma that was resolved using Gelfoam particles and coils in the left L4 lumbar artery. While L4 did not show active extravasation, the presence of vasospasm as well as the patient’s improvement in symptoms following the procedure make it highly likely that this artery was the one damaged during LP. The patient’s body habitus and challenges in attaining CSF access during LP lend further evidence to this. In such cases, IR embolization is very important in preventing further bleeding and associated complications.

## Conclusion

Urgent IR embolization is an important method of resolving active or potentially active sites of bleeding within the body. This can be especially important when such causes of bleeding are associated with iatrogenic damage in failed procedures. While LP-associated retroperitoneal hematomas are an extremely rare complication, they can be devastating if not detected and managed appropriately.

## Author contributions

The authors declare that this is their work, and they all approve the content of this manuscript. They confirm that this manuscript has not been published previously, in any language, in whole or in part, and is not currently under consideration elsewhere.

## Ethical clearance

This project did not involve any research and no ethical clearance was required.

## Patient consent

Radiology uses images which are exempted from consent.
